# Antimicrobial resistance and prevalence of *Listeria* species from raw milk and dairy products in Burdur, Turkey

**DOI:** 10.1002/vms3.1551

**Published:** 2024-07-25

**Authors:** Zeki Erol, Zübeyde Polat, Ali Soyuçok, Halil Yalçın, Fulya Taşçı

**Affiliations:** ^1^ Department of Food Hygiene and Technology, Faculty of Veterinary Medicine Necmettin Erbakan University Ereğli, Konya Turkey; ^2^ Department of Food Hygiene and Technology, Faculty of Veterinary Medicine Burdur Mehmet Akif Ersoy University Burdur Turkey; ^3^ Department of Food Processing, Burdur Food Agriculture and Livestock Vocational School Burdur Mehmet Akif Ersoy University Burdur Turkey

**Keywords:** antimicrobial‐resistant, dairy products, food safety, *Listeria*, milk

## Abstract

**Objectives:**

Worldwide, but especially in emerging nations, concerns about food safety pose a serious obstacle to societal and economic progress. This research aimed to examine the prevalence of *Listeria* spp. in raw milk and dairy products in Burdur, as well as the presence of genes associated with biofilm formation and antibiotic resistance in the isolates.

**Methods:**

A total of 185 samples, including raw milk, curd, cream, butter, yogurt and cheese, were randomly collected in Burdur. The enrichment and isolation methods specified by the United States Department of Agriculture was used to identify *Listeria* species in milk and dairy product samples. Culture‐positive strains were identified as *Listeria* genus and as species by PCR. Antibiotic susceptibility of the isolates was evaluated against 14 antibiotics using the disc diffusion technique (EUCAST).

**Results:**

Of them, 2.2% (4/185) were positive for *Listeria* spp. *Listeria* species were isolated from cheese and yogurt samples. Two of them were *Listeria* innocua 1.1% (2/185), one was *Listeria ivanovii* 0.5% (1/185) and the other was *Listeria* welshimeri 0.5% (1/185). As a result of multiplex PCR of the biofilm genotypic marker *luxS* and *flaA* genes, the flaA gene was detected in three of four isolates, the *luxS* gene was detected in one isolate, and these two genes were not found in one isolate. Although all isolates were resistant to gentamicin and rifampicin, they also showed multidrug resistance.

**Conclusion:**

This study revealed that the diversity of prevalence of *Listeria* spp. in Burdur requires microbial risk assessment in the milk and dairy products value chain and the need to focus on the problem of multiple antibiotic resistance.

## Graphical abstract

Graphical Abstract: No evidence of *Listeria monocytogenes* was found in Burdur's public bazaars’ raw milk or dairy products. Some strains of *Listeria* were isolated from other dairy products (cheese and yogurt) other than raw milk in the Burdur public bazaars. Some of the *Listeria* spp. were associated with genes effective in biofilm formation. Although all isolates resisted gentamicin and rifampicin, they also exhibited multidrug resistance.

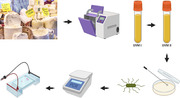

## 1 INTRODUCTION

*Listeria* are Gram‐positive bacteria that are extensively distributed in a variety of environments and areas. Twenty species of *Listeria*, including the recently discovered *Listeria thailandensis* sp. nov, consist of the genus *Listeria* (Leclercq et al., 2019). They are grouped under two groups as *Listeria sensu stricto* and *Listeria sensu lato* (Orsi & Wiedmann, 2016). *Listeria monocytogenes*, *Listeria ivanovii* and *Listeria seeligeri*, which are in the group of *L. sensu stricto*, were stated to be in the pathogenic group. *L. monocytogenes* is the most common species linked to disease in both animals and humans. In addition, human infections with *L. seeligeri* and *L. ivanovii* have been rarely reported (Allerberger & Wagner, 2010; Rees et al., 2017). Infections and functional diversity between isolates are mostly determined by pathogenicity islands, which are clusters of virulence factors. Host cell adhesion or attachment, invasion, polymerization and cell‐to‐cell dissemination are all facilitated by *Listeria* Patogenity Island‐1 (LIPI‐1) (*actA*, *prfA*, *hlyA*, *mpl*, *plcA* and *plcB*) and LIPI‐2 (*inlA*, *inlB*, *inlC* and *inlJ*) genes in listeriosis (Radoshevich & Cossart, 2018; Wang et al., 2018). It has been shown that both *L. ivanovii* and *L. seeligeri* share the virulence gene cluster of *L. monocytogenes* (Gouin et al., 1994). Nonpathogenic *Listeria innocua*, *Listeria welshimeri* and *Listeria grayi* lack the virulence gene cluster in its entirety (Vázquez‐Boland et al., 2001). Furthermore, *Listeria* is the ethiological agent of listeriosis, a potentially fatal illness in newborns, the elderly, pregnant women and others with impaired immune systems. Listeriosis is often associated with mild symptoms such as non‐invasive febrile gastroenteritis or severe symptoms such as septicaemia, meningitis and abortion, with a significant risk of mortality (Erol & Taşçı, 2024; Ewida et al., 2022; Tasci et al., 2010, 2024). Compared to other foodborne disease, such as campylobacteriosis (0.03%) and salmonellosis (0.22%), listeriosis is among the most dangerous due to the high fatalities documented due to the disease (Sołtysiuk et al., 2022).

One of the best ways to treat severe or protracted bacterial infections in people or animals is with antibiotic therapy. Antimicrobial drugs are becoming less effective as more and more organisms develop resistance to them (Ağaoğlu & Tutun, 2022; Tutun & Yurdakul, 2023). Antibiotics from the beta‐lactam group, such as penicillin and ampicillin (AM), are often used either alone or in combination with gentamicin (CN) for the treatment of listeriosis. Meta‐analysis studies show that antibiotics used or recommended in the treatment of listeriosis are reported to be the most resistant antibiotics (Cufaoglu et al., 2021; Khademi & Sahebkar 2019; Mpundu et al., 2021).

The bacteria belonging to the genus *Listeria* have a broad distribution in many natural environments and are also found in diverse dietary sources. *Listeria* spp. may enter the food chain through environmental contamination. Foods such as raw vegetables, fruits, meat, fish, poultry and raw milk, as well as their processing environments, may be contaminated with *Listeria* spp. and serve as a vehicle for the spread of *Listeria* spp. (Erol & Taşçı, 2021; Midam et al., 2023; Shantha & Shubha Gopal, 2014). Dairy products are typically consumed either fresh or after being processed, including through fermentation. Although fermentation generates an acidic environment that can deter many pathogens, *L. monocytogenes* can occasionally survive, particularly when the initial contamination level is substantial (Siddiqui et al., 2023). One of the most prominent foods linked to human listeriosis outbreaks is contaminated dairy products. Cases continue to occur as a consequence of post‐processing contamination and ingestion of raw milk products, despite the fact that milk pasteurization has decreased the risk (Matto et al., 2018). Especially, *L. monocytogenes* is a danger to dairy products since it can survive and develop at refrigeration temperatures in the presence of moderate salt concentrations and neutral pH activity (Rugji & Dinçoğlu, 2022; Rugji et al., 2023). *L. monocytogenes* was detected in 0.51% for all ready to eat milk products (*N* = 26.154) in the European Union. This incidence was 0.69% for cheeses (*N* = 14.985) and 0.30% for milk (*N* = 1585) (European Food Safety Authority [EFSA], 2022). Fifty cases of listeriosis related to dairy products were reported in the USA in 2022 (Centers for Disease Control and Prevention [CDC], 2023). In Turkey, the presence of *L. monocytogenes* in milk and dairy products is regulated in Annex‐1 of the Turkish Food Codex Microbiological Criteria Regulation. According to Annex‐1, the presence of *L. monocytogenes* is not allowed in 5 × 25 g or mL milk and dairy products (Resmi Gazete [RG], 2011). In this respect, the practice is in line with the USA (United States Department of Agriculture [USDA], 2022). The situation in the European Union is a bit complicated. Similarly, the 5 × 25 g or mL rule applies. However, there are situations where up to 100 CFU per g or mL is permitted (EUR‐Lex, 2005).

Burdur is known as the “Netherlands of Turkey” due to its position in animal husbandry. There are approximately 192 thousand cattle and 357 thousand sheep and goats in Burdur, and 383 thousand tons of milk are produced annually (Anon, 2023). Dairy products in Burdur are produced in small production centres or in the community. These products are also sold unpackaged. It is important to determine the actual status of *Listeria* spp. in milk and dairy products in Burdur. This study aims to determine possible *Listeria* spp. in milk and dairy products sold in Burdur and its surroundings, to identify genes related to biofilm formation, and to evaluate antimicrobial resistance profile.

## 2 MATERIALS AND METHODS

### 2.1 Samples collection

Samples were obtained from public bazaars and open sales points in the centre and districts of Burdur. A total of 185 dairy products, including 28 raw milk, 10 curd, 10 cream, 15 butter, 33 yogurt and 89 cheese, were collected from June to August 2023. Each sample was placed in a sterilized container under refrigeration conditions and transported to the laboratory for analysis.

### 2.2 Isolation and identification of *Listeria*

The enrichment and isolation methodology suggested by the USDA was used to identify *Listeria* species in food samples (Mcclain & Lee, 1988). Aseptically, 25 g of each sample (cheese, curd, butter, yogurt and cream) and 25 mL of milk were taken and mixed for 2 min in 225 mL of *Listeria* enrichment broth (UVM I, Quelab) and incubated at 37°C for 24 h. After adding 1 mL of pre‐enriched culture to 9 mL of UVM II (Frazer broth, Quelab), it was incubated at 37°C for 24 h. After streaking the enriched broth onto Agar *Listeria* according to Ottaviani and Agosti and incubating it at 37°C for 48 h, the plates were examined for the presence of *Listeria* colonies. Biochemical tests were performed on all of the isolates to confirm that they belonged to the genus *Listeria*. These included the catalase test, gram staining, the motility test at 25°C and the acid production from rhamnose and xylose.

### 2.3 PCR assays

Positive suspected strains obtained by the cultural method were first confirmed as *Listeria* genus and then as *Listeria* species by PCR. The *flaA* gene, which helps flagella adhere to surfaces, and the *luxS* gene, which supports biofilm development, were also studied. The GeneJET Genomic DNA Purification Kit (Thermo Scientific, K0722) was used to extract DNA, and the process was carried out in accordance with the manufacturer's instructions. The primers used in the PCR test, primer sizes and PCR conditions are given in Table 1. The following protocol was used to perform simplex PCR on a total volume of 20 µL: In a PCR tube, we combined 10 µL of 2X PCR master mix (Ruby Tag Master, Jena Bioscience, PCR‐164L), 3 µL of the DNA template, 0.5 µL of each primer (F or R) and 6 µL of nuclease‐free water. In multiplex PCRs, nuclease‐free water was reduced by the amount of the primers added to complete the 20 µL volume.

**TABLE 1:** In the process of PCR analysis, genes, primer sequences and PCR conditions used.

Primer and size (bp)	Primer sequence (5′ → 3′)	PCR conditions	References
*mono A‐B* (371 bp)	F: CAAACTGCTAACACAGCTACT R: GCACTTGAATTGCTGTTATTG	Denaturation 94°C, 45 s Annealing 55°C, 30 s Extention 72°C, 10 min (simplex)	Bubert et al. (1992)
*Iap* (1400 bp)	F: GCTACAGCTGGGATTGCGGT R: TTATACGCGACCGAAGCCAA	Denaturation 94°C, 60 s Annealing 56°C, 45 s Extention 72°C, 45 s (simplex)	Bubert et al. (1997)
*Iva1* (1112 bp)	F: CTACTCAAGCGCAAGCGGCAC R: 5‐TTATACGCGACCGAAGCCAAC	Denaturation 95°C, 30 s Annealing 62°C, 30 s Extention 72°C, 90 s (simplex)	Bubert et al. (1999)
*Sel1* (1099 bp)	F: TACACAAGCGGCTCCTGCTCAAC R: TTATACGCGACCGAAGCCAAC		
*Wel1* (1048 bp)	F: CCCTACTGCTCCAAAAGCAGCG R: TTATACGCGACCGAAGCCAAC		
*Ino2* (1017 bp)	F: ACTAGCACTCCAGTTGTTAAAC R: TTATACGCGACCGAAGCCAAC	Denaturation 94°C, 45 s Annealing 62°C, 60 s Extention 72°C, 45 s (simplex)	
*flaA* (363 bp)	F: GGAAATGCCAGCGCTACACTCTTT R: ATTGCATGCAGGAACTTCTGTCGC	Denaturation 94°C, 30 s Annealing 58°C, 30 s Extention 72°C, 7 min (multiplex)	Warke et al. (2017)
*luxS* (208 bp)	F: GCGCAAGAACGTTTAGCATCTGGT R: TTGAGTAGCAGCACCTGTAGCAGT		

### 2.4 Antibiotic test

The disc diffusion technique was used to assess the isolates’ antimicrobial susceptibility (European Committee on Antimicrobial Susceptibility Testing [EUCAST], 2021). A total of 14 antibiotics were utilized, representing 10 distinct classes. *Listeria* spp. isolates cultured the night before were looped and suspended in sterile tubes containing 5 mL of 0.85% physiological saline solution and adjusted to 0.50 McFarland (10^8^ CFU/mL) turbidity with a McFarland densitometer (Biosan). Subsequently, the suspension that had been generated was introduced onto Mueller Hinton agar (MHA) (Oxoid 337) supplemented with 5% defibrinated horse blood and 20 mg/L β‐NAD (Sigma‐Aldrich, N6522) by the process of streaking a sterile cotton swab over the agar's surface. Following this, the antibiotic discs were positioned on the agar and allowed to dry at room for 5 min. After that step, MHA was incubated at 37°C for 18 h. Following incubation, zone diameters were measured. The assessment of zone diameters was carried out according to two international organisations. The assessment that was carried out in accordance with EUCAST (2019) took into account the breakpoint tables of *L. monocytogenes* for erythromycin (E), meropenem (MEM) and co‐trimoxazole (STX) antibiotics; *Staphylococcus aureus* for tetracycline, ciprofloxacin (CIP), CN, rifamycin (RA), chloramphenicol and ofloxacin (OFX) antibiotics; and *Enterobacterales* for amoxicillin–clavulanic acid antibiotics. On the other hand, the breakpoint tables of *Enterococcus* spp. for AM, penicillin (P) and vancomycin antibiotics and *Enterobacteriaceae* for streptomycin (S) antibiotics were taken into account in the assessment that was carried out in accordance with the Clinical and Laboratory Standards Institute (Clinical and Laboratory Standards Institute [CLSI], 2017).

### 2.5 Statistical analysis

In the statistical analysis, Minitab (version 21.4.1) was used. In the comparison of prevalence of *Listeria* spp. Ficher’ exact test was performed 95% confidence interval (95%). Statistical significance level was defined as *p* < 0.05.

## 3 RESULTS

**FIGURE 1:**
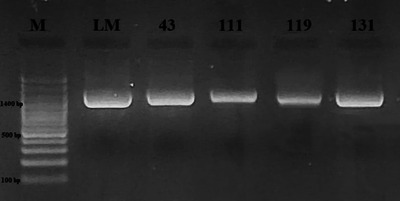
Electrophoresis gel image of the *iap* (1400 bp) gene region (Left to right; M: 3 kb DNA Marker (100 bp) LM: *Listeria monocytogenes* ATTC 13932, 43‐111‐119‐131: *Listeria* spp.).

**FIGURE 2:**
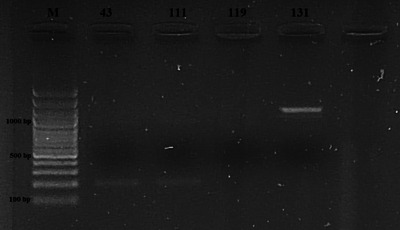
Electrophoresis gel image of the *Iva1* (1112 bp) gene region (Left to right; M: 3 kb DNA Marker (100 bp), 131: *Listeria ivanovii*).

**FIGURE 3:**
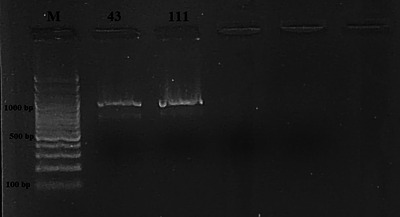
Electrophoresis gel image of the *Ino2* (1017 bp) gene region (Left to right; M: 3 kb DNA Marker (100 bp), 43‐111: *Listeria innocua*).

**FIGURE 4:**
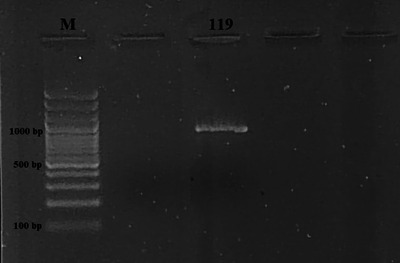
Electrophoresis gel image of the *Wel1* (1048 bp) gene region (Left to right; M: 3 kb DNA Marker (100 bp), 119: *Listeria welshimeri*).

Four samples of milk and dairy products were found positive for *Listeria* spp. by culture method and then confirmed by PCR (Figure 1). The presence of *Listeria* spp. was detected in three samples of cheese and one sample of yogurt, but no *Listeria* spp. was detected in samples of milk, butter, cream and cream samples. There was no significant difference between cheese, yogurt, milk, butter, cream and curd samples in terms of *Listeria* spp. (*p* > 0.05). The overall prevalence in all milk and milk products was 2.2% (4/185). Table 2 presents the findings about the occurrence of *Listeria* spp. and *Listeria* species in the milk and dairy product samples that were subjected to analysis. PCR findings are also presented in Figures 2, 3, 4. On the other hand, as a result of multiplex PCR of *luxS* and *flaA* genes, *flaA* gene was found in three out of four isolates, *luxS* gene was detected in one isolate and these 2 genes were not found in one isolate.

**TABLE 2:** Prevalence of *Listeria* spp. in milk and dairy products in Burdur.

	N	n (%)	Lm	Lin	Ls	Lw	Liv	k
Cheese	89	3 (3,4)	–	2	–	1	–	3
Yogurt	33	1 (3)	–	–	–	–	1	1
Milk	28	–	–	–	–	–	–	–
Butter	15	–	–	–	–	–	–	–
Crema	10	–	–	–	–	–	–	–
Curd	10	–	–	–	–	–	–	–
Total	185	4 (2,2)	–	2	–	1	1	4

Abbreviations: k, *Listeria* spp number from one or more positive samples; Lm, *Listeria monocytogenes*; Lin, *Listeria innocua*; Ls, *Listeria seeligeri*; Lw, *Listeria welshimeri*; Liv, *Listeria ivanovii*; N, number of samples analysed, n, number of *Listeria* spp. positive samples.

The antimicrobial resistance profiles of four isolates, which were verified as *Listeria* spp. using PCR, were investigated (Table 3). Overall, all *Listeria* isolates were resistant to two or more antimicrobial agents. All isolates were resistant to CN and RA. Except isolate 43, all other isolates were resistant to erythromycin. All isolates also showed multidrug resistance.

**TABLE 3:** Profiles of antibiotic resistance in *Listeria* spp.

	Antimicrobial agents													
Isolates	AMC 30 µg	C 30 µg	CN 10 µg	MEM 10 µg	CIP 5 µg	AM 10 µg	VA 30 µg	E 15 µg	TE 30 µg	P 10 U	STX 25 µg	OFX 5 µg	RA 5 µg	S 10 µg
43	S	S	R	S	S	S	S	S	S	S	S	S	R	S
111^a^	S	S	R	S	S	S	S	R	S	S	S	S	R	I
119^a^	S	S	R	R	R	S	S	R	S	S	R	R	R	R
131^a^	S	S	R	S	S	S	S	R	S	S	R	R	R	R

Abbreviations: AMC, amoxicillin–clavulanic acid; C, chloramphenicol; CN, gentamicin; CIP, ciprofloxacin; MEM, meropenem; RA, rifamycin; STX, co‐trimoxazole; TE, tetracycline.

^a^ Multidrug resistance, 43 (*Listeria innocua*), 111 (*L. innocua*), 119 (*Listeria welshimeri*) and 131 (*Listeria ivanovii*).

## 4 DISCUSSION

The occurrence of dairy contamination may be seen as a complex phenomenon influenced by several factors. Contamination can occur in raw milk, at the farm level, or in the cheese processing environment (Melo et al., 2015). In addition, contamination after low pasteurisation temperature and processing should not be ignored. Apart from this, dairy products produced from raw milk are very likely to be part of this process (Rugji & Dinçoğlu, 2022). In this study, as mentioned above, *Listeria* spp. were isolated from 4 (2.2%) milk and dairy product (raw milk [0], curd [0], cream [0], butter [0], yoğurt [1] and cheese [3]) samples out of 185 samples analysed (Table 2). These findings align with the outcomes reported by Moshtaghi and Mohamadpour (2007) but *Listeria* spp. contamination levels were found to be lower in previous studies (Seyoum et al., 2015; Shamloo et al., 2015; Şanlıbaba et al., 2018) than in the present study. However, no *L. monocytogenes* were detected. In contrast, other *Listeria* species were isolated from cheese and yogurt samples in this study. Two of them were *L. innocua* (43–111) 1.1% (2/185), one was *L. ivanovii* (131) 0.5% (1/185) and the other was *L. welshimeri* (119) 0.5% (1/185). Although *L. ivanovii* infections are not as common as *L. monocytogenes* infections, they nonetheless result in the loss of fetuses, stillbirths and preterm deliveries in ruminants. *L. ivanovii* infections have also been found in people on very rare occasions, where they predominantly cause diarrhoea, bacteraemia in immunocompromised persons and foetal loss in pregnant women (Guillet et al., 2010). Although *L. innocua* does not cause disease in people, it is similar to *L. monocytogenes* and responds similarly to it in terms of chemical and physical stressors as well as biofilm formation (Rosa et al., 2022). *L. welshimeri* is not pathogenic due to the lack of virulence genes in LIPI‐1, which is located in the chromosomal locus between *prs* and *Idh* involved in *Listeria* pathogenesis (Hain et al., 2006). Despite the fact that the prevalence of *Listeria* in dairy products may vary, it has been demonstrated that *Listeria* strain is prevalent in samples of milk and dairy products. In this study, the highest prevalence of *Listeria* was found in cheese samples. The predominant isolate was also *L. innocua*. The prevalence of *Listeria* species has been reported in various studies. In one study, *Listeria* was detected in 11.7% of the samples. *L. monocytogenes* was found in 2.4% of the samples, *L. innocua* in 5.6% and *L. seeligeri* in 1.8%. The highest prevalence was reported in raw milk and cheese samples (Akrami‐Mohajeri et al., 2018). In another study conducted in Turkey, the overall prevalence of *Listeria* spp. in homemade cheese samples (142) was found to be 33.1%. Out of 142 samples, 13 (9.2%) had *L. monocytogenes*. *L. innocua* (9.2%), *L. seeligeri* (5.6%), *L. grayi* (4.9%), *L. ivanovii* (2.1%) and *L. welshimeri* (2.1%) were also found (Arslan & Özdemir 2008). A research in Egypt found *Listeria* spp. in 24 (10%) of 240 dairy product samples. *Listeria* species were found in fresh cream, ice cream, buttermilk cheese and Kareish cheese at 6 (15%), 8 (20%), 5 (12.5%) and 5 (12.5%), respectively, but not in Talaga cheese and yogurt. *L. grayii* (45.83%) was the most common, followed by *L. monocytogenes* (33.33%), *L. welshime*ri (12.51%) and *L. innocua* 2 (8.33%) (Elshinaway et al., 2016). In a study in Iran, 21 (7.14%) of 292 samples were positive for *Listeria* spp. *Listeria* spp. was found in 5.91 (5.49%), 12.63 (19.04%), 3.27 (11.11%) and 1.25 (4%) of samples of raw milk, ice cream, cream and frank, respectively. *Listeria* were not found in yogurt, butter, cheddar and cheese. The species most often identified *was L. innocua*, accounting for 16.21 (5.44%) of the isolates. This was followed by *L. monocytogenes*, which constituted 4.21 (19%) of the isolates, and *L. seeligeri*, which accounted for 1.21 (4.7%) of the isolates (Shamloo et al., 2015). *Listeria* spp. prevalence study findings may vary depending on the nature of the tested food or the methodology used to detect it.

*flaA* and *luxS* are two pivotal genes involved in the process of biofilm formation (Finn et al., 2023). It has been shown that flagellar expression is necessary for optimal bacterial adhesion, colonization and invasion (Duan et al., 2013). Flagellar motility is present in all *Listeria* species. The *flaA* gene encoding the *Listeria* flagellin protein is important for genotypic identification (Gorski, 2008). The *luxS* gene encodes the *S*‐ribosylhomocysteinase enzyme *S*‐ribosylhomocysteinase for autoinducer‐2, an important part of quorum sensing responsible for important mechanisms such as growth, motility, adhesion and biofilm formation (Finn et al., 2023; Vendeville et al., 2005). Except for one cheese sample isolate, *flaA* or *luxS* gene region was detected in all isolates. Only the yogurt sample isolate had both gene regions. Warke et al. (2017) detected the presence of both genes in 23 of 38 *L. monocytogenes* isolates from food, clinical cases and cattle farm environments. One isolate was positive only for the *flaA* gene. In another biofilm research, 14 of 25 *L. monocytogenes* isolates had *luxS* and 18 had *flaA* (56% and 72%, respectively). Nine (36%) food isolates had the luxS gene, whereas five (20%) water and food isolates had the *flaA* gene. Both *flaA* and *luxS* genes were found in nine isolates (five food and four water) (Kaptchouang Tchatchouang et al., 2022). In this study, the presence of *flaA* and *luxS* genes genotypically supports the possible production of biofilms.

The public health concern of foodborne microorganisms that are becoming more resistant to antibiotics has received increased focus in recent years. Several *Listeria* strains have shown resistance to drugs; this is particularly worrisome for humans as antibiotic treatment is required for more severe cases of listeriosis. *Listeria spp*. resistance to antimicrobial medications widely used to treat listeriosis is becoming more of an issue, despite the guidelines offered. A considerable number of these strains are multidrug‐resistant (Cufaoglu et al., 2021; Mpundu et al., 2021). In the present study, all four isolates were susceptible to penicillin groups (AM and P), the first choice antibiotics. Previous research by Sołtysiuk et al. (2022), Korsak et al. (2012) and Babacan (2021) also showed that all strains of *L. innocua* and *L. monocytogenes* from bulk tank milk and all strains of *L. monocytogenes*, *L. innocua* and *L. ivanovii* isolated from cow milk were susceptible to AM. On the other hand, reports of penicillin group resistance at both high and low levels have been made by some authors. Skowron et al. (2019) found that penicillin resistance was the most prevalent among *Listeria* isolates obtained from milk, accounting for 44.4% of isolates. Srinivasan et al. (2005) observed a very high resistance rate of 92% among *L. monocytogenes* strains isolated from dairy farms against AM. Rosa et al. (2022) detected lower resistance levels in *L. innocua* strains to P (11.8%) and AM (11.8%) in dairy processing chain. AM specifically targets the *Listeria* penicillin‐binding proteins PBP1 and PBP2, effectively inhibits PBP3 and PBP4, and has a lesser affinity for PBP5 (Vicente et al., 1990). Though it is possible that mutations in susceptible PBPs could raise the minimum inhibitory concentration of penicillins, and transposon mutagenesis seems to produce AM‐resistant mutants, these mutants have PBPs that have not been changed, and they lose their resistance when they are incubated at high temperatures, so the target for AM resistance is still unknown (Poroś‐Głuchowska et al., 2003; Poroś‐Głuchowska & Markiewickz, 2003). In contrast, the yogurt sample isolate (*L. welshimeri*) exhibited resistance to the second‐choice antibiotics (MEM, E and STX), whereas one cheese sample isolate (*L. ivanovii*) showed resistance to E and STX. A similar antibiotic resistance was reported by Sołtysiuk et al. (2022). The loss of the bactericidal synergy between trimethoprim and sulfamethoxazole due to resistance to trimethoprim created resistance to cotrimoxazole, the first line of defence against *Listeria* infections. Genes that encode trimethoprim‐resistant dihydrofolate reductases (*dfrD* or *dfrG*) or housekeeping *dhfr* gene mutations can lead to acquired resistance. These mutations give high or low amounts of resistance, respectively (Charpentier et al., 1999). Clinical and food isolates have relatively low resistance to E (less than 3%) (Baquero et al., 2020). Both previous studies and the current study found the resistance of *Listeria* species to E to be higher than 3% (Akrami‐Mohajeri et al., 2018; Rahimi et al., 2010, 2012; Sarker & Ahmed, 2015; Sołtysiuk et al., 2022). Antimicrobial resistant genes (ARGs) are typically encoded on mobile genetic elements (White et al., 2002). ARGs resistance to erythromycin may be based on the previously reported possibility of plasmid‐mediated (Poyart‐Salmeron et al., 1992) or transfer from lactic acid bacteria in fermented milk (Toomey et al., 2009). Interestingly, all isolates exhibited resistance to CN, which is used in combination with AM to treat listeriosis. CN resistance has been documented in previous reports as either low or susceptible (Akrami‐Mohajeri et al., 2018; Jorgensen et al., 2021). The presence of the efflux pump, which provides resistance to benzalkonium chloride, has been linked to a particular decrease in *Listeria* CN sensitivity (Rakic‐Martinez et al., 2011). Another antibiotic to which all isolates were resistant was RA. In contrast to the present study, RA resistance of *Listeria* spp. was shown to be 6.9%, 3.5% and 2.1% in isolates from ready‐to‐eat foods, product processing plants and dairy products, respectively (Akrami‐Mohajeri et al., 2018; Arslan & Özdemir, 2020; Jorgensen et al., 2021). RA has a wide range of antibacterial activities because it specifically targets the RNA polymerase β‐subunit in its mode of action. It has antimicrobial action against both mycobacteria and Gram‐positive bacteria (Ruiz‐Bolivar et al., 2011). *RpoB* mutations, namely in the gene that codes for the β‐subunit of RNA polymerase, often result in resistance to (Chenal‐Francisque et al., 2014). Meanwhile, 75% of all tested isolates (*L. welshimeri*, *L. ivanovii* and one strain of *L. innocua*) were described as multidrug resistant. *L. welshimeri* showed the highest multidrug resistance, exhibiting resistance to eight (CN, MEM, CIP, E, STX, OFX, RA and S) antibiotics. It was followed by *L. ivanovii* and *L. innocua* with six (CN, E, STX, OFX, RA and S) and 3 (CN, E and RA), respectively. In the last years, there has been a considerable increase in the level of concern around the growing resistance to a number of different classes of antibiotics.

## 5 CONCLUSION

As a result, the current study found *Listeria* spp. to be 2.2% in yogurt and cheese sold in the public bazaar in Burdur. However, important limitations of the research were the inconsistency in the range of products available at points of sale and the unwillingness of some vendors to provide samples. This prevented homogeneous distribution in the samples. This has affected the comparison between products. On the other hand, *L. monocytogenes* was not among the isolates detected, but other *Listeria* species, *L. ivanovii*, *L. innocua* and *L. welshimeri*, were isolated from yogurt and cheese samples. The presence of important marker genes for biofilm was revealed in these species. These isolates were resistant to eight classes of antibiotics, and multidrug resistance was a significant finding. For this reason, in Burdur, comprehensive microbial risk assessment and examination of antimicrobial resistance status in the milk value chain, starting from raw milk production, are of great importance to ensure food safety.

## AUTHOR CONTRIBUTIONS

**Zeki Erol; Zübeyde Polat**: Conceptualization; data curation; formal analysis; investigation; methodology; visualization; writing—original draft. **Ali Soyuçok; Halil Yalçın and Fulya Taşçı**: Conceptualization; data curation; formal analysis; methodology; visualization; writing—original draft.

## CONFLICT OF INTEREST STATEMENT

The authors declare no conflicts of interest.

## FUNDING INFORMATION

The authors declare that no funding was received for this research.

### ETHICS STATEMENT

Ethical approval was not required as this article does not contain research data on live animals.

### PEER REVIEW

The peer review history for this article is available at https://publons.com/publon/10.1002/vms3.1551.

## Data Availability

Data is available on request.
